# Direct tuning of graphene work function via chemical vapor deposition control

**DOI:** 10.1038/s41598-020-66893-y

**Published:** 2020-06-18

**Authors:** Taegeun Yoon, Qinke Wu, Dong-Jin Yun, Seong Heon Kim, Young Jae Song

**Affiliations:** 1 SKKU Advanced Institute of Nanotechnology (SAINT), Sungkyunkwan University (SKKU), Suwon, 16419 Korea; 2 Shenzhen Geim Graphene Center (SGC), Tsinghua-Berkeley Shenzhen Institute (TBSI), Tsinghua University, Shenzhen, 518055 PR China; 3 Analytical Engineering Group, Samsung Advanced Institute of Technology, Suwon, 16678 Korea; 4 Department of Physics, Myongji University, Yongin, 17058 Korea; 5 Department of Nano Engineering, Sungkyunkwan University (SKKU), Suwon, 16419 Korea; 6 Department of Physics, Sungkyunkwan University (SKKU), Suwon, 16419 Korea

**Keywords:** Materials science, Nanoscience and technology, Physics

## Abstract

Besides its unprecedented physical and chemical characteristics, graphene is also well known for its formidable potential of being a next-generation device material. Work function (WF) of graphene is a crucial factor in the fabrication of graphene-based electronic devices because it determines the energy band alignment and whether the contact in the interface is Ohmic or Schottky. Tuning of graphene WF, therefore, is strongly demanded in many types of electronic and optoelectronic devices. Whereas study on work function tuning induced by doping or chemical functionalization has been widely conducted, attempt to tune the WF of graphene by controlling chemical vapor deposition (CVD) condition is not sufficient in spite of its simplicity. Here we report the successful WF tuning method for graphene grown on a Cu foil with a novel CVD growth recipe, in which the CH_4_/H_2_ gas ratio is changed. Kelvin probe force microscopy (KPFM) verifies that the WF-tuned regions, where the WF increases by the order of ~250 meV, coexist with the regions of intrinsic WF within a single graphene flake. By combining KPFM with lateral force microscopy (LFM), it is demonstrated that the WF-tuned area can be manipulated by pressing it with an atomic force microscopy (AFM) tip and the tuned WF returns to the intrinsic WF of graphene. A highly plausible mechanism for the WF tuning is suggested, in which the increased graphene-substrate distance by excess H_2_ gases may cause the WF increase within a single graphene flake. This novel WF tuning method via a simple CVD growth control provides a new direction to manipulate the WF of various 2-dimensional nanosheets as well as graphene.

## Introduction

Since the first exfoliation of a single graphene layer from highly oriented pyrolytic graphite (HOPG), graphene has been considered as one of the most promising materials for next generation of electronic devices, due to its outstanding optical, electrical, and mechanical properties^1–3^. For applications such as transparent electrodes, tuning the work function (WF) of graphene is essential since the mismatch of the energy levels between the graphene electrode and materials in the active layers would degrade the performance of devices^4^. In particular, when graphene makes a contact with other semiconductors or metals in electronic or optoelectronic devices, the WF difference between them determines the band structure alignment in the interface and the contact characteristics, i.e. whether it is Ohmic or Schottky^5–8^. Therefore, it is significant to control the WF of graphene in graphene-based devices.

Lots of efforts have been made for tuning the WF of graphene^9–15^. A major approach to tune the graphene WF is surface engineering, particularly by depositing a very thin layer (about one layer) of alkali metal, such as Cs, Li, Sr, or Ba, that are sometimes combined with a proper amount of oxygen^12^. Another approach is by the doping of graphene due to a lack of dangling bonds and surface states^9–15^. Although a great deal of meaningful results of graphene WF tuning have been reported by doping or surface engineering, this approach has a limitation because basically it needs an additional process in the device fabrication, which increases the fabrication cost and time. Thus, a simple WF tuning method for large-area graphene film without additional fabrication process is highly desirable.

Here we report the WF tuning method for graphene grown on a Cu foil with a controlled CVD growth recipe, in which the CH_4_/H_2_ gas ratio is simply controlled. This method does not add any additional process to a routine CVD graphene growth process. Raman and X-ray photoelectron spectroscopy (XPS) confirmed that the graphene grown with the CVD recipe of high H_2_ gas portion has considerably increased WF, while its chemical structure is almost same as that of a conventional graphene. Moreover, Kelvin probe force microscopy (KPFM) verifies that the WF-tuned regions, in which WF increases by the order of ~250 meV, coexist with the regions of intrinsic WF within a single graphene flake. It is also demonstrated that the WF-tuned area can be manipulated by pressing it with an atomic force microscopy (AFM) tip and the tuned WF returns to the intrinsic WF of graphene. This novel WF tuning method via a simple CVD growth control provides a new direction to manipulate the electronic properties of graphene and various two-dimensional analogues.

### Experiment

Two kinds of graphene samples were synthesized through low-pressure chemical vapor deposition (LPCVD) on a Cu foil, by varying a volumetric velocity ratio of CH_4_ to that of H_2_ from 40:10 to 40:40. The growth process was conducted for 20 to 40 minutes to ensure enough coverage of graphene. The quality of graphene on Cu foils was identified by Raman spectroscopy (XperRam 200, Nanobase). XPS experiments were conducted to investigate the chemical structure and the work function of the graphene samples by using an ultrahigh vacuum (UHV) XPS instrument (PHI 5000 VersaProbe, ULVAC-PHI) with a Ar gas cluster ion beam (GCIB) sputtering equipment.

To obtain the spatial variation of WF for the two kinds of graphene on Cu, we also performed KPFM, which is an AFM based technique to acquire WF information via measuring contact potential difference (CPD) for every pixel. A map of CPD, the relative surface potential (SP) of an AFM tip to that of a sample, is simultaneously obtained with a topographic image of the sample surface. The AFM tip for KPFM is generally metallic or metal-coated since measurement of CPD requires at least one conductive component. In addition, manipulation on graphene surfaces and graphene-copper interfaces was done by lateral force microscopy (LFM), a contact mode AFM technique which can characterize or modify tribological properties of the sample surface. All AFM-based measurements were conducted with a commercial atomic force microscope (NX-10, Park Systems Corp.). KPFM measurements involved electrically grounding graphene samples, through which SP of any sample may have the same zero point.

## Results & Discussion

**Figure 1:**
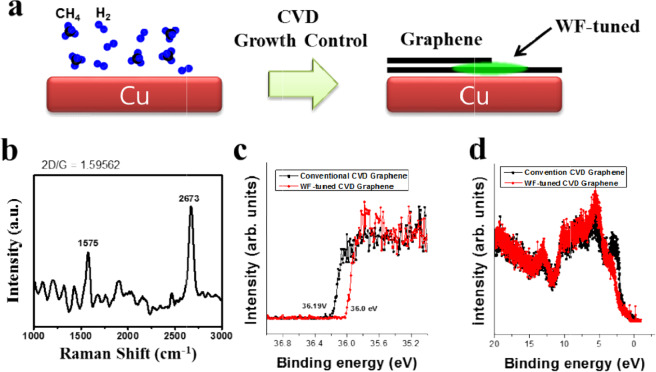
(**a**) CVD growth process for WF-tuned graphene on Cu foil. (**b**) Raman spectroscopy result for WF-tuned graphene. (**c**) XPS secondary cutoff and (**d**) valence band offset plots for a conventional and a WF-tuned graphene.

As shown in Fig. 1a, we synthesized graphene on a Cu foil by controlling CH_4_ to H_2_ ratio in a LPCVD method. The conventional graphene was grown with the CH_4_:H_2_ ratio of 40:10 and the ratio was changed to 40:40 for WF-tuning by the higher H_2_ content. Figure 1b shows the Raman spectroscopy result for the graphene synthesized with the new CVD recipe (CH_4_:H_2_ = 40:40). In the Raman spectrum, the characteristic G and 2D peaks of graphene are clearly measured at 1575 and 2673 cm^−1^, respectively. The most common method to determine the layer number of graphene is to calculate the intensity ratio of G and 2D peaks in Raman spectrum^16–19^. For the graphene sample grown with the new recipe, the intensity ratio of 2D/G was ~1.6, which corresponds to monolayer or bilayer graphene, thinner than trilayer^16–19^. Other commonly used method to estimate the layer number of graphene is to measure the full width at half maximum (FWHM) of 2D peak. The FWHM of the 2D peak in Fig. 1b is ~50 cm^−1^ and it corresponds to the monolayer or bilayer graphene, which is in a good agreement with the estimation by 2D/G calculations. Based on the Raman spectroscopy analysis, it was confirmed that the graphene grown by the new CVD recipe is same as a conventional CVD-grown graphene on a Cu foil, in terms of its intrinsic properties.

To investigate the WF properties of the graphene grown on a Cu foil with the new CVD recipe, we performed XPS, a representative surface analysis technique to explore the electronic structure including WF. Figure 1c,d show a secondary cutoff and valence band offset spectra for graphene samples synthesized with conventional and new CVD recipes. In Fig. 1c, the secondary cutoff value is 36.0 and 36.19 eV for the graphene sample grown by the new recipe and a conventional one, respectively. The WF values can be calculated by subtracting the secondary cutoff values from our X-ray beam energy (40.8 eV). Based on the simple calculation, the WF value of the graphene grown by the new CVD recipe is ~4.8 eV and that of the conventional graphene is ~4.61 eV. It is notable that the graphene grown by the new recipe has ~200 meV higher than WF comparing with the conventional graphene. In conclusion, our new CVD recipe, in which the only change is a simple increase of H_2_ content, successfully tunes the WF of graphene. In addition, to check the chemical structure of the WF-tuned graphene by the new CVD recipe, we performed XPS depth profiling measurements by using a mild Ar GCIB sputtering technique, which is known as the most powerful technique to sputter organic sample surfaces without a severe chemical damage^20,21^. Figure S1 shows the XPS depth profile results for a conventional and the WF-tuned graphene samples. In Fig. S1, the chemical structure of the WF-tuned graphene is almost same as that of a conventional graphene, except that the WF-tuned graphene sample shows higher oxidation of Cu.

**Figure 2:**
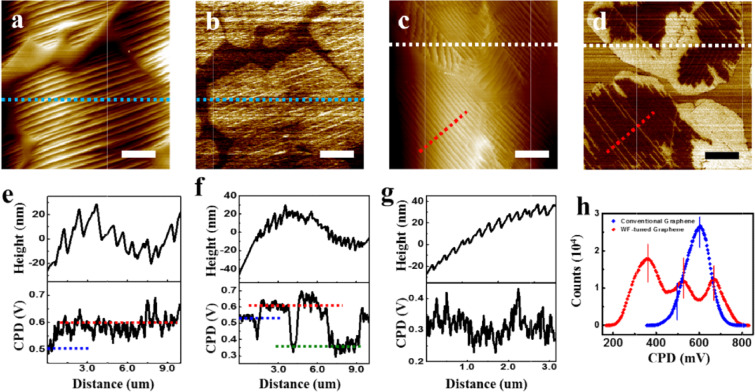
(**a**) AFM topographic and (**b**) the corresponding KPFM images for a conventional graphene grown on Cu foil. (**c**) AFM topographic and (**d**) the corresponding KPFM images for a WF-tuned graphene grown on Cu foil. (**e**) Line profiles extracted from the dashed blue lines in (a) and (b). (**f**) Line profiles extracted from the white dashed lines in (c) and (d); (**g**) Line profiles extracted from the red dashed lines in (c) and (d). (h) CPD distributions of (b) and (d). All scale bars are 2 μm.

Although XPS is a reliable method to measure WF of graphene, it has a limit because its lateral resolution of ~10 μm is not sufficient to examine the spatial variation of WF on a graphene surface. For this purpose, we performed KPFM that is a useful AFM-based technique to image the spatial variation of WF on surfaces of various materials at the nanoscale. Figure 2 shows the KPFM results for a convention and a WF-tuned graphene. The CPD images of a conventional and a WF-tuned graphene are clearly different in Fig. 2b,d, while there is no significant difference in their topographic images (Fig. 2a,c). As shown in Fig. 2b,e (the line profile extracted from the dotted line in Fig. 2b), the SP within the graphene area is almost homogeneous, while the graphene area is evidently distinguishable from a Cu surface due to its ~100 meV higher SP, *i.e*. ~100 meV lower WF, than that of Cu. On the contrary, besides the graphene area is still clearly distinguishable from a Cu surface by the difference of SP in Fig. 2d, the prominent inhomogeneity is also observed within a single graphene island. The dark regions, not shown in Fig. 2b, are newly observed within single graphene flakes. The WF of the dark region in the CPD map is higher than that of the bright region, because WF is in the converse relation with SP. According to the CPD line profiles (Fig. 2f.g) extracted from the dotted lines in Fig. 2d, the CPD difference between bright and dark graphene regions is ~250 meV, which can be compatible with the value of ~200 meV acquired in XPS. In Fig. 2f, the dotted blue, red, and green lines represent the SP levels for a Cu substrate, bright and dark graphene regions, respectively, and therefore the WF of the dark graphene region increased by ~250 meV with the new CVD recipe. The variation of WF in the WF-tuning process is also shown in the CPD distribution plots for the conventional and the WF-tuned graphene samples in Fig. 2h. The blue colored curve in Fig. 2h, which indicates the CPD distribution for the conventional graphene acquired from Fig. 2b, shows one main peak that represents the homogenous SP of graphene area. The peak for a Cu substrate was merged with the main peak as its left side shoulder. On the other hand, the red colored curve in Fig. 2h, indicating the CPD distribution of the WF-tuned graphene acquired from Fig. 2d, clearly shows three peaks that correspond to a Cu substrate, intrinsic, and WF-tuned graphene.

From our XPS and KPFM results, the WF-tuned graphene samples, synthesized with the new CVD recipe of a higher H_2_ gas portion, show the increased WF by the order of ~250 meV and this increment is significantly high in terms of WF tuning. However, although the graphene WF tuning by our new CVD recipe is indubitable, its mechanism is still ambiguous. Here, we examine four possible cases of graphene WF variation. The first one is that the WF tuning may originate from the formation of a bilayer structure within a single graphene flake. It is known that, at a high H_2_ pressure, the (second) adlayer graphene is grown beneath the (first) top layer, *i.e*. between the first layer and the Cu surface and variation in the number of layers can change the WF of graphene^22,23^. However, the formation of bilayer structure cannot explain our WF increase of ~250 meV, because WF tends to decrease as the number of graphene layers increases^23,24^. The second possible origin of our WF-tuning is the local curvature induced by the ripples within a single domain graphene flake, which may be formed by excessive H_2_ gases in the new CVD process. This hypothesis also cannot elucidate the WF increase in our WF-tuned graphene, because it was reported that the curvature increase on graphene ripples leads to the gradual decrease of WF, although its tendency is not monotonous^24^. The third possibility is that the doping of graphene, in particular, hydroxylation, happens in the new CVD process. In the literature, the functionalization of graphene by hydroxyl (-OH) groups significantly increases the WF of graphene^13^. However, the difference in hydroxylation between our conventional and WF-tuned graphene samples is not severe in the XPS results (Fig. S1a). In addition, it is unconvincing that a macroscopic gas control in the CVD process induces the abrupt local change in doping within a single graphene layer. Lastly, the fourth possible case is that the distance between graphene and a Cu surface may increase by excessive H_2_ gases. Khomyakow *et al*. reported the relationship between the graphene WF and the graphene-substrate distance for various metal substrates including Cu (111)^25^. According to the literature, as the distance between graphene and a Cu (111) surface increases from the equilibrium distance of ~3.3 Å, the WF of graphene also monotonously increases by ~ 380 meV up to the distance of ~4.0 Å and it is almost saturated for longer distances^25^. This monotonous increase and saturation of WF with increasing graphene-substrate distance can explain the feature of our WF-tuned graphene samples.

**Figure 3:**
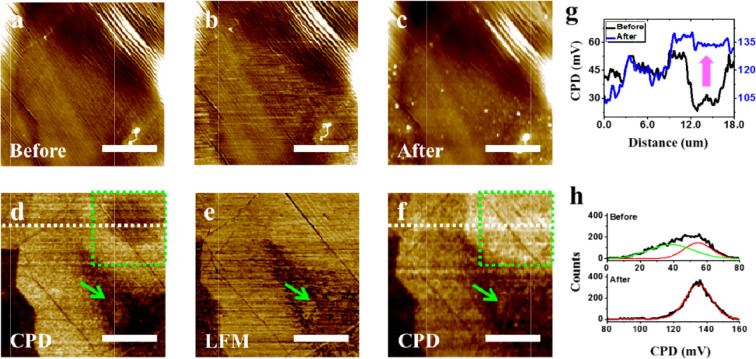
(**a**) Non-contact AFM topography and (d) the corresponding KPFM images of WF-tuned graphene. (**b**) Contact AFM topography and (e) the corresponding LFM images acquired on the same area of the WF-tuned graphene sample. (**c**) Non-contact AFM topography and (**f**) the corresponding KPFM images acquired on the same area of the WF-tuned graphene sample after LFM measurement. (g) Line profiles extracted from the white dashed lines in (**d**) and (f). (h) CPD histograms of the green square regions in (d) and (f). All scale bars are 6 μm.

**Figure 4:**
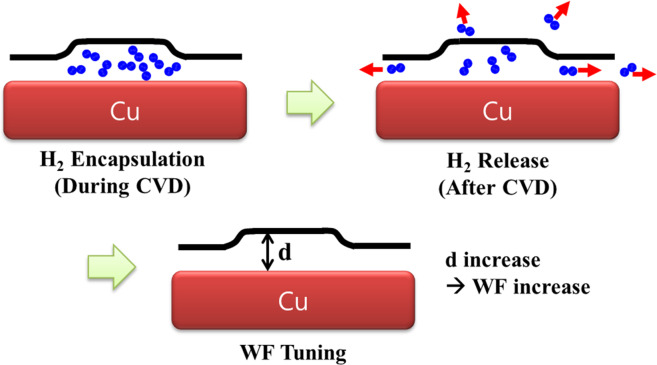
Schematic diagram of WF-tuning mechanism by the CVD growth control.

For the experimental verification of the mechanism of our WF-tuning method, we performed LFM on the WF-tuned graphene sample in Fig. 3. LFM is a useful technique to measure the tribology properties of sample surfaces^26,27^. Because the LFM is operated in a contact mode AFM, it can be used to modify the distances between two-dimensional materials and substrates. Figure 3a,d show the topographic and the corresponding CPD images of the WF-tuned graphene acquired before the LFM measurement. In Fig. 3d, two WF-tuned regions, (*i.e*. dark regions), are indicated by a green arrow and a green dotted square. Figure 3b,e show the topographic and LFM images successively acquired on the same sample area. One notable thing is that the WF-tuned region indicated by the green arrow in Fig. 3d shows different characteristics in the LFM image in Fig. 3e, which implies that the WF-tuned region has different tribology properties induced by different graphene-substrate interaction. After the LFM measurement, we performed the KPFM measurement again on the same sample area. Figure 3c,f show the topographic and the corresponding CPD images acquired after the LFM measurement. A clear change is observed in the region indicated by the green square. The dark region of increased WF inside the green square in Fig. 3d disappeared in Fig. 3f. The return of WF is obviously shown by comparing two CPD line profiles in Fig. 3g, which were extracted from the dotted lines in Figs. 3d,f. Figure 3h shows the histograms of CPD distributions in the green square regions in Fig. 3d,f. While the CPD distribution in the green square region was broad and can be fitted with two Gaussian functions before the LFM measurement, it changed to a narrow peak which can be fitted with only one Gaussian function after the LFM measurement. From the combined LFM and KPFM experiments, it was demonstrated that the tuned WF returned to its intrinsic value by pressing the graphene surface using an AFM tip during the LFM measurement. Therefore, the fourth hypothesis for the mechanism of our WF-tuning method, *i.e*. the WF-tuning originates from the increase of graphene-substrate distance within a single graphene domain, is highly plausible. The detailed process for our suggested WF-tuning mechanism is depicted in Fig. 4. The surplus H_2_ gases are encapsulated beneath a graphene in the CVD process and the graphene-substrate distance slightly increases on the H_2_ encapsulated area. Then, the H_2_ gases are released through graphene defects or the side gap between graphene and Cu substrate after the CVD process. Finally, the increased graphene-substrate distance which increases the WF maintains.

## Conclusions

In this study, we developed a novel WF tuning method for graphene grown on a Cu foil simply by changing the CH_4_:H_2_ gas ratio in a CVD growth. While the WF-tuned graphene does not have any significant difference in a chemical structure comparing with a conventional graphene in Raman spectroscopy and XPS measurements, it shows the considerably increased WF. In addition, it was demonstrated that the WF-tuned regions, in which the WF increases by the order of ~250 meV, coexist with the regions of the intrinsic WF within a single graphene flake in Kelvin probe force microscopy (KPFM). In particular, it was possible to manipulate the WF-tuned graphene area by pressing it with an AFM tip and return the WF to its intrinsic value by the combination of KPFM and LFM. Furthermore, we suggest a highly plausible WF tuning mechanism for the new CVD process, based on the combined KPFM and LFM experiment. In the suggested WF tuning mechanism, the graphene-substrate distance increases by encapsulation of excess H_2_ gases beneath a graphene flake and it gives rise to the increased graphene WF. This study provides an experimental work on the modulation of graphene WF and the WF-tuning method by controlling the graphene-substrate interface could be applied to alternative two-dimensional analogues. This novel WF tuning technique suggests a new concept in the modulation of electronic properties of various two-dimensional analogues as well as graphene.

## Supplementary information

Supplementary Information.
