# Supplementary material for: Direct tuning of graphene work function via chemical vapor deposition control
Source: Sci Rep. 2020 Jun 18;10:9870. doi: 10.1038/s41598-020-66893-y (PMC7303148; doi:10.1038/s41598-020-66893-y)
Supplement: Supplementary file 1 — Supplementary Information. [file 41598_2020_66893_MOESM1_ESM.docx]

**Supporting Information**

**Direct tuning of graphene work function via chemical vapor deposition control**

Taegeun Yoon^†,1^, Qinke Wu^†,2^, Dong-Jin Yun^3^, Seong Heon Kim^*,4^, Young Jae Song^*,1,5,6^

^1^SKKU Advanced Institute of Nanotechnology (SAINT), Sungkyunkwan University (SKKU), Suwon, 16419, Korea

^2^Shenzhen Geim Graphene Center (SGC), Tsinghua-Berkeley Shenzhen Institute (TBSI), Tsinghua University, Shenzhen 518055, PR, China

^3^Analytical Engineering Group, Samsung Advanced Institute of Technology, Suwon, 16678, Korea

^4^Department of Physics, Myongji University, Yongin 17058, Korea

^5^Department of Nano Engineering, Sungkyunkwan University (SKKU), Suwon, 16419, Korea

^6^Department of Physics, Sungkyunkwan University (SKKU), Suwon, 16419, Korea

^†^TY and QW contributed this work equally.

^*^Corresponding authors: shkim@mju.ac.kr (SHK), yjsong@skku.edu (YJS)


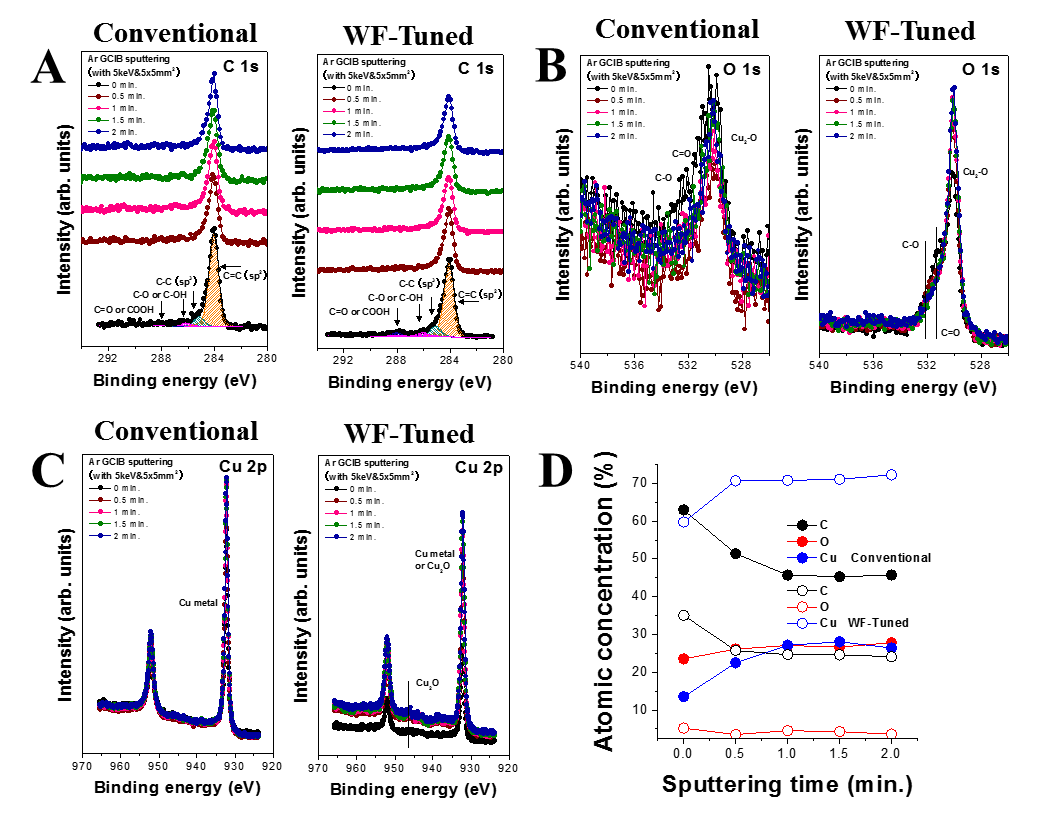


Figure S1. XPS depth profile results by Ar GCIB sputtering for a conventional and a WF-tuned graphene. (a) C 1s, (b) O 1s, (c) Cu 2p, and (d) atomic concentration.
